# Supplementary material for: Discovery and pharmacophoric characterization of chemokine network inhibitors using phage-display, saturation mutagenesis and computational modelling
Source: Nat Commun. 2023 Sep 16;14:5763. doi: 10.1038/s41467-023-41488-z (PMC10505172; doi:10.1038/s41467-023-41488-z)
Supplement: Supplementary file 4 — Source Data [file 41467_2023_41488_MOESM4_ESM.zip › SourceData/scripts/xNNKs_20230808183658.tar!/xNNKs/analysis/boxplots/SV_HD2X_isoleucine_boxplot.pdf]

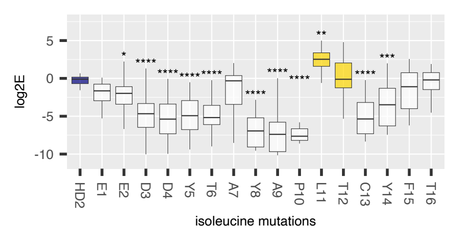

log2E
5
0
-5
-10
*
****
****
****
****
****
****
**
****
***
HD2
E1
E2
D3
D4
Y5
T6
A7
Y8
A9
P10
L11
T12
C13
Y14
F15
T16
isoleucine mutations
